# Parasites

**Published:** 1872-10

**Authors:** 


### PARASITES,

Meaning "feeding places near at hand," are living things, vegetable and animal, which live upon other living things until their life is all exhausted, when they die themselves, as the Spanish moss which dresses the forests of the lower Mississippi in funereal gloom, and the tape-worm of the human body. There are three upon the living exterior man, but only when he grovels in uncleanliness, squalor, and filth,—the head louse, the crab louse, and the body louse.

### THE LOUSE

is the special designation of the animal which infests the hair of the human head; it does not live in the hair of any other part of the body, as under the arms, over the breast, etc. The feet, on their three pairs of legs, are all alike. Their eggs stick fast to the hair, and are called

### NITS.

In six days the young one is hatched from this nitty egg, and in eighteen days begins to lay eggs itself, averaging about fifty; thus one egg becomes twenty-five hundred living lice in about six weeks; hence, nothing short of total extermination will effect their riddance. Black hair easily shows the eggs, which sometimes line the whole length of the hair. The itching is caused by their diving their heads into the skin or scalp to get something to eat; and as scratching, to remove this itching, wounds and irritates the tender scalp, it is not uncommon among poor, neglected children, to find the whole scalp a mass of ugly, inflamed sores, which exude a fluid, and even blood comes from the wounds of the finger-nails; this fluid and blood harden, making altogether a mass of matted hair, and blood, and filth, horrible to contemplate. This exuded fluid makes food for them to grow to a large size, and propagate in immense numbers; so large, indeed, that they cannot pasture on one head; then they wander away in search of others, by the help of their six legs; hence it is very easy for one lousy child to people the heads of a whole school in a short time. In this way accidents will sometimes happen in the best regulated families; for even sitting by the side of an infected person for a moment or two, or handling a hat or shawl, endangers infection; and as they propagate rapidly, the most careful may find a whole brood living upon their very life-blood before they are aware of it. But a million of them, nits and all, can be murdered in a minute, by getting an ounce of

COCCULUS INDICUS, steeped, mashed or pounded, in a wine-glass of alcohol for a day or two; then dampen the head with warm water, put a tea-spoonful in the palm of the hand and rub it faithfully over the hair and scalp, so that every hair and every pin-point of the scalp shall be saturated with it, and by the time you are done, the murdered million can be combed away. This is certainly preferable to chasing each particular individual until caught, and then smashing it with the thumb-nail, which is very tedious, and unless a person has great skill and a sharp eyesight, to take every prisoner, the whole work would have to be done again; but the tincture of the *cocculus indicus*, used sometimes to kill fishes, is effectual, and is as perfectly harmless to the scalp as common spirits alone.

To impress this on the reader's mind, it may be stated that this cure of lousiness came to the knowledge of the writer at an expense of over a thousand dollars, little thinking that after the lapse of many years, the investment would be returned with interest in the propagation and perpetuation of the knowledge in the form of a book. It is said to be quite as effectual in removing lice from herds of cattle and cats and poodle dogs; it is worth the experiment. It has no effect on other vermin, such as chinches and fleas, except to make them very lively or dead drunk for a short time, when they get up and scamper off with the utmost agility. The louse has infected the heads of almost all nations, from remote antiquity; dried nits have been found in the hair of the oldest mummies.

THE CRAB LOUSE

infests the hair of another portion of the body; it takes hold of a hair, runs down it, until it seeks the skin, where it deposits the nits. It lives on human blood, as does the musquito, and its bite causes a fierce itching. It is curious to note that these two insects never interfere with each other's pasturage. The crab louse claims to itself every hairy part of the body, even to the eyelashes and eyebrows, but it never invades the scalp; it however claims the whiskers, while the other is content to come down to them, but never infests them. This animal infests old clothing, and especially the berths of ships; hence many cleanly persons find themselves infested after being a few days on board ship. The tincture above named is sometimes efficacious in their destruction.

THE BODY LOUSE

is much like that of the head, only larger; its home is in the seams and creases of the clothing, but it feeds on the blood of the parts, especially of those made tender by the creases, and which not only make the skin at those points juicy, but afford better places for hiding. Although the tincture named will kill these vermin, perhaps the best use to be made of such clothing is to boil or burn it. The body of a person may not show a single parasite, and yet the clothing will swarm with them; it is wearing clothing a long time without change or washing which harbors these creatures, and gives them time to multiply in incredible numbers. With strict personal cleanliness, no one need fear of being infected with any of these vermin; neglect and filth and laziness, or helplessness, promote their multiplication.

MERCURIAL OINTMENT, if well rubbed in, night and morning, will give eventual riddance, but this sometimes salivates. An ointment made of sulphur, used freely, will destroy them eventually; a level tea-spoonful of flowers of sulphur to a level table-spoon of hog's lard.

STAVEACRE SEED, soaked in vinegar, is a good remedy, or made into an ointment. Sometimes the plentiful use of sweet oil to the scalp and hairy parts is effectual; or *sabadilla* seed, ground; six parts mixed with one of hog's lard. When the hair is in a matted condition from long neglect, cut it close off, and use the means named. If there are scabs and sores, procure a free condition of the bowels in addition to the other means, eating lightly of coarse breads and fruits; these are cooling, and help to keep down inflammation, and remove irritation. If the hair is long, and there is an aversion to losing it, wash it with alcohol or vinegar twice a day, rubbing it in well, so as to reach every hair and every part of it, and every pin-point of the scalp, or other parts infected; this will procure complete riddance.

CAPUCHIN POWDER

is used in some countries, made of equal parts of the powder of the seeds of *cocculus indicus*, *staveacre*, and *sabadilla;* but that of the cocculus alone, mixed with lard and used as an ointment, is sufficient; but as all ointments are objectionable because they gather dust, the powder of the cocculus may be sufficient.

It is almost impossible sometimes to prevent sailors' bunks, soldiers' barracks, prisons, hospitals, and large public schools, from infection; of all the soldiers, North and South, in the great civil war, perhaps not over one in a thousand escaped infection, officers not excepted, thus making it a subject well worthy of the space given to it.

